# Association between current e-cigarette use and chronic obstructive pulmonary disease: a meta-analysis focusing on exclusive e-cigarette users

**DOI:** 10.3389/fpubh.2026.1802572

**Published:** 2026-04-22

**Authors:** Yanqiu Jiang, Yao Wang, Mengya Yang

**Affiliations:** Clinical Research Center, The Quzhou Affiliated Hospital of Wenzhou Medical University, Quzhou People’s Hospital, Quzhou, China

**Keywords:** COPD, e-cigarettes, public health, respiratory health, tobacco smoking

## Abstract

**Background:**

While often promoted as a safer alternative to traditional cigarettes, electronic cigarettes (e-cigarettes) are linked to uncertain long-term health effects. Their independent relationship with chronic obstructive pulmonary disease (COPD) remains unclear, mainly due to the confounding effects of traditional cigarette use. This meta-analysis aimed to evaluate the association between current e-cigarette use and the prevalence of COPD. As a key secondary aim, we conducted a subgroup analysis focusing on exclusive e-cigarette users—individuals who currently use e-cigarettes but have never smoked traditional cigarettes—to preliminarily explore the independent association.

**Methods:**

A comprehensive literature search was conducted in PubMed, Embase, Web of Science, the Cochrane Library, China National Knowledge Infrastructure, and Wanfang Database for studies published up to 12 November 2025. Eligible observational studies that assessed the association between e-cigarette use and the prevalence of COPD were included, and pooled estimates were calculated using a random-effects model.

**Results:**

A total of 15 studies were included in the meta-analysis. Current e-cigarette use was associated with a significantly higher odds of having COPD (pooled odds ratio [OR] = 2.03, 95% confidence interval [CI]: 1.61–2.56). Former e-cigarette users also had an elevated association with COPD (OR = 1.82, 95% CI: 1.38–2.41). Subgroup analyses indicated the prevalence of COPD was higher among exclusive e-cigarette users (OR = 2.09, 95% CI: 1.46–3.00), dual users of e-cigarettes and traditional cigarettes (OR = 3.13, 95% CI: 2.01–4.87), and current e-cigarette users with a smoking history (OR = 2.17, 95% CI: 1.41–3.35). Sensitivity analysis supported the robustness of these findings, and no evidence of publication bias was observed.

**Conclusion:**

This meta-analysis suggests that current e-cigarette use is associated with a higher prevalence of COPD. This association remained evident among exclusive e-cigarette users, indicating a potential link independent of traditional smoking history.

**Systematic review registration:**

https://www.crd.york.ac.uk/PROSPERO/view/CRD420251218716, identifier (CRD420251218716).

## 1 Introduction

Chronic obstructive pulmonary disease (COPD) presents a major public health challenge. According to data from the Global Burden of Disease (GBD), approximately 212.3 million people worldwide are living with COPD, which poses a serious threat to respiratory health globally (1–3). Currently, COPD is ranked as the fourth leading cause of death globally, resulting in approximately 3.5 million deaths in 2021 and accounting for approximately 5% of all global deaths (4). Previous studies have demonstrated that COPD exerts a substantial burden on global economic development, with total economic losses estimated to reach as high as $4.33 trillion (5). However, the global prevalence of COPD varies considerably, influenced by factors such as geographic region, environmental exposure, and individual risk profiles. In the general population, the prevalence of COPD among adults aged over 30 years is approximately 10.3%, whereas individuals in high-risk groups, including smokers and those with occupational or environmental exposure, face a markedly increased risk (6).

Electronic cigarettes (e-cigarettes) were introduced in the early 21st century as electronic aerosol-generating devices that produce inhalable aerosols by heating a liquid (7). E-cigarettes were initially designed to assist smokers in reducing or quitting traditional tobacco use by mimicking the behavioral and sensory aspects of smoking (8). Unlike traditional cigarettes, e-cigarettes do not involve combustion and therefore do not generate tar or many of the harmful by-products present in conventional tobacco smoke (9). Consequently, when e-cigarettes are used as a complete alternative to combustible cigarettes, they may reduce the risk of certain smoking-related diseases, including lung cancer and cardiovascular disease. As a result, the use of e-cigarettes has increased rapidly worldwide, particularly among adolescents and young adults, who represent the fastest-growing user group (10, 11). However, e-cigarette aerosols are not without risks; they contain multiple constituents that may adversely affect the respiratory system, including ultrafine particles, volatile organic compounds, and other toxic chemicals known to cause airway irritation (12). These findings have raised increasing concerns regarding the potential long-term health effects of e-cigarette use, especially regarding pulmonary and respiratory outcomes (13, 14).

Given the chronic and progressive nature of COPD, early identification of modifiable risk factors is essential for effective disease prevention and management. Although e-cigarettes are widely perceived as a less harmful alternative to traditional smoking, their long-term health effects remain poorly understood, particularly among never-smokers. This uncertainty underscores the need for rigorous investigation into whether e-cigarette use may independently increase the risk of COPD or contribute to disease development and progression. This study primarily aims to investigate the association between current e-cigarette use and the prevalence of COPD. Additionally, as a critical and pre-specified subgroup analysis, we focus on exclusive e-cigarette users—individuals who currently use e-cigarettes but have never smoked traditional cigarettes—to minimize the confounding effects of smoking history and to preliminarily explore the potential independent association. The insights gained will help inform public health policies and support informed clinical decision-making.

## 2 Methods

### 2.1 Study design

We conducted a systematic review and meta-analysis to gather and evaluate the literature on the association between e-cigarette use and the prevalence of COPD. The review complied with the guidelines set by the Preferred Reporting Items for Systematic Reviews and Meta-Analyses (PRISMA) (Supplementary Table S1) (15). The protocol for this study was also registered with the International Prospective Register of Systematic Reviews (PROSPERO) (CRD420251218716).

### 2.2 Data sources and search strategy

We systematically searched English databases, such as PubMed, Embase, Web of Science, and the Cochrane Library, and Chinese databases, such as China National Knowledge Infrastructure (CNKI) and Wanfang Patent Database (WFPD). The search period spanned from the establishment of these databases to 12 November 2025. The start date was not restricted to ensure that all relevant studies published before the specified search date were included. The search strategy used a combined approach of MeSH terms and free-text terms. No language or document type restrictions were imposed. The complete search strategy (including all keywords and filter conditions) is detailed in Supplementary Table S2.

### 2.3 Eligibility criteria

To be included in the study, the following criteria must be met;

1. Studies must investigate the association between e-cigarette use and the prevalence of COPD.
2. Participants should be 18 years of age or above and must clearly specify their e-cigarette use status (current use, former use, or never use).
3. Studies must provide quantitative risk estimates such as odds ratios (ORs), risk ratios (RRs), or sufficient data to calculate these metrics.
4. Studies must be published in peer-reviewed journals.

The diagnostic criteria for COPD included self-reporting diagnoses, pulmonary function tests (e.g., spirometry), and electronic health records (EHRs). Although self-reported COPD diagnoses may exhibit some inherent variability, they were included in the current study to ensure comprehensiveness, given the limited number of available studies. The primary focus was on exposure factors, specifically e-cigarette use, with the outcome measure being patients diagnosed with COPD.

Exclusion criteria included case reports, conference abstracts, editorials, and review articles. Studies that focused exclusively on respiratory diseases without specific data on COPD were also excluded.

### 2.4 Study selection

To determine eligibility for inclusion, two reviewers independently reviewed the abstracts and titles of each retrieved study. Subsequently, the same group of reviewers obtained and reviewed the full texts of studies considered potentially relevant. Any disputes regarding study inclusion were resolved through discussion or, when necessary, by involving a third reviewer. To enhance efficiency and reduce manual errors, we utilized EndNote for the elimination of duplicate literature, study screening, and label annotation, thereby improving workflow consistency.

### 2.5 Data extraction and quality assessment

Data were collected through structured extraction tables. The collected data included important details of the study, such as authors, year of publication, journal name, study title, country of origin, study design, data sources, study duration, total sample size, subject characteristics (such as age and gender), the definition of e-cigarettes usage (current/former use), the definition of the control group (never smoker), the effect size and corresponding 95% confidence interval (95% CI), the diagnostic criteria for COPD (self-report/pulmonary function tests/EHRs), and the way of obtaining smoking history (self-report/urine nicotine test). The data extraction was completed by two independent reviewers, and any disputes were resolved through discussion. If necessary, a third reviewer would be consulted. Using the Newcastle–Ottawa Scale (NOS), two evaluators independently assessed the quality of the included studies. The studies were evaluated based on subject selection, study group comparability, and identification of the association between e-cigarette exposure and COPD outcomes (Supplementary Table S3). All studies evaluating e-cigarette users in this meta-analysis adjusted the age factor in the analysis to ensure that the observed association between e-cigarette use history and COPD prevalence was not affected by age-related effects.

### 2.6 Data synthesis and statistical analysis

Data analysis was performed using R software version 4.5.2 to investigate the association between e-cigarette use and the prevalence of COPD. To account for inter-study heterogeneity, a random-effects meta-analysis was used. Results were presented as pooled ORs and 95% CI. Inter-study heterogeneity was assessed using the *I*^2^ statistic, where 25, 50, and 75% represented low, moderate, and high heterogeneity, respectively (16). Although ORs were the primary pooled measure in the meta-analysis, some included studies also reported RR. Despite their correlation, these two measures have distinct interpretations: ORs describe the odds of an outcome occurring, while RRs indicate the relative risk of that outcome. Given the epidemiological interpretation differences between ORs and RRs, and the potential for additional assumptions introduced by mathematical transformations in studies with high heterogeneity, we did not perform a uniform effect size conversion. Instead, we categorized the included studies into two datasets based on study types: cross-sectional studies and prospective cohort studies, for subgroup analysis. This approach enables us to evaluate the consistency of association patterns while acknowledging that effect estimates from different designs may vary numerically due to inherent characteristics of the measures. To ensure the robustness of the results, a leave-one-out sensitivity analysis was conducted to prevent individual studies from disproportionately influencing the pooled effect size (17). This sensitivity analysis also evaluated the consistency of results across different study designs and quality levels. In all analyses, *p*-values of <0.05 were considered statistically significant.

## 3 Results

### 3.1 Literature search

**Figure 1:**
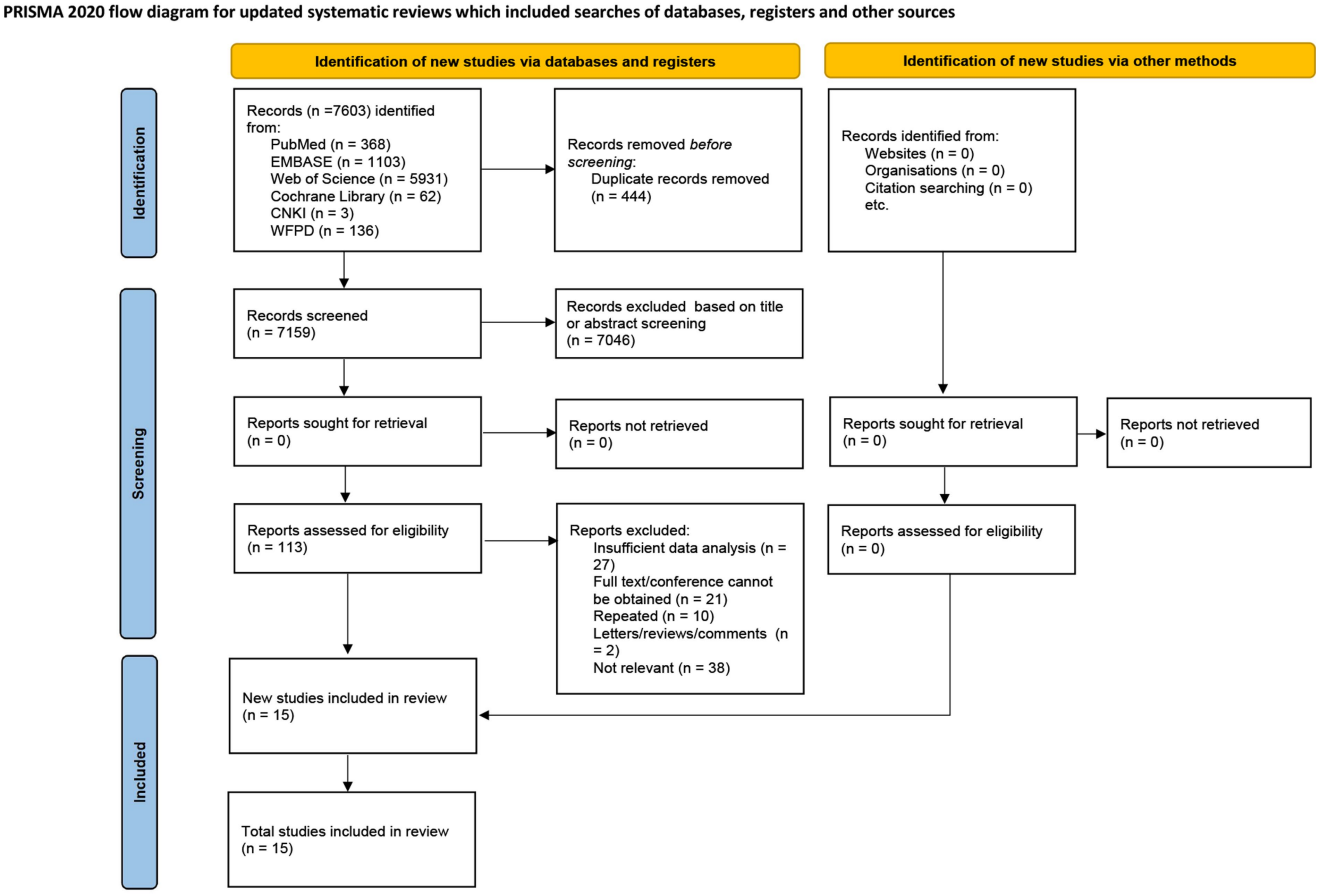
Flow diagram of study identification, screening, and inclusion process.

Detailed information on systematic literature retrieval is shown in Figure 1. A total of 7,603 records were retrieved through database searches, including 368 from PubMed, 5,931 from Web of Science, 1,103 from Embase, 62 from the Cochrane Library, 3 from CNKI, and 136 from WFPD. After excluding 444 duplicate entries, 7,159 records were screened. Based on the titles and abstracts, 7,046 non-compliant articles were excluded, leaving 113 full-text articles for detailed review. Comprehensive analysis revealed that 98 articles did not meet the inclusion criteria due to irrelevance, inability to obtain full-text versions, or insufficient data analysis. Ultimately, 15 studies were selected for inclusion in the systematic review (18–32).

### 3.2 Characteristics of included studies

The included studies were conducted in the United States (13 studies), China (1 study), and South Korea (1 study), with 12 cross-sectional studies and 3 cohort studies (Table 1). The study population comprised general adult participants aged 18 to over 50 years, with females accounting for 25 to 60% of participants. COPD diagnosis relied on self-report in 12 studies, pulmonary function tests (e.g., FEV1/FVC < 0.70) in 2 studies, and EHR data in 1 study. The confirmation of smoking history relied on self-report. The effect values of e-cigarette users are mainly reported as ORs or RRs, and the study sample size varies widely, ranging from a small cohort of approximately 6,945 participants to a large-scale study of 1,927,541 participants.

**Table 1:** Characteristics of included studies.

Author, year	Country	Study design	Diagnostic criteria	Sample size (total)	Participants’ age (years)	Female	Study period	RR or OR (current e-cigarette user)
Antwi (2022) (18)	United States	Cross-sectional	Self-report	177,209	≥18	86,816	2018	1.47 (1.22–1.76)
Bircan (2021) (19)	United States	Cross-sectional	Self-report	8,736	≥18	3,008	2016–2018	1.44 (1.42–1.46)
Burns (2025) (20)	United States	Cross-sectional	Self-report	22,997	≥40	12,138	2020	2.84 (1.69–4.80)
Comiford (2024) (21)	United States	Cross-sectional	Self-report	1,927,541	≥18	996,539	2016–2018, 2020–2021	1.40 (1.34–1.47)
Kim (2025) (22)	Korean	Cross-sectional	FEV1/FVC < 0.70	19,356	57.7	10,801	2013–2014	2.57 (1.82–3.64)
Paulin (2022) (23)	United States	Prospective cohort	Self-report	6,945	≥40	NA	2014–2019	1.85 (1.25–2.74)
Osei (2020) (24)	United States	Cross-sectional	Self-report	705,159	≥18	376,732	2016–2017	1.77 (1.28–2.45)
Song (2024) (25)	China	Prospective cohort	FEV1/FVC < 0.70	10,326	20–55	3,599	2015–2020	1.15 (1.01–1.31)
Wills (2019) (26)	United States	Cross-sectional	Self-report	8,087	≥18	3,772	2016	3.59 (2.68–4.81)
Xie (2020) (27)	United States	Cross-sectional	Self-report	891,242	≥18	445,621	2016–2017	3.83 (3.53–4.15)
Perez (2019) (28)	United States	Cross-sectional	Self-report	32,320	≥18	8,254	2013–2014	1.47 (1.21–1.79)
Goldberg Scott (2023) (29)	United States	Cross-sectional	EHRs	119,593	≥18	72,228	2015–2019	2.16 (1.77–2.63)
Wills (2022) (30)	United States	Cross-sectional	Self-report	214,945	≥18	111,597	2020	1.63 (1.47–1.81)
Xie (2020) (31)	United States	Prospective cohort	Self-report	21,618	≥18	10,601	2013–2018	1.57 (1.15–2.13)
Cordova (2022) (32)	United States	Cross-sectional	Self-report	26,072	≥18	13,557	2013–2018	5.82 (4.06–8.36)

### 3.3 Quality assessment of included studies

The quality of included studies was assessed using the NOS scale (Supplementary Table S3). The NOS scores ranged from 3 to 6. Only one study was rated as low quality (score of 3), while the remaining studies were of medium quality (scores of 4–6). To examine the potential influence of study quality on the pooled results, a sensitivity analysis was performed using a threshold NOS score of 5. This threshold was chosen to create a balanced comparison: studies with a score ≥5 were considered at least moderate quality, whereas those with a score ≤4 were classified as lower quality. This distinction helps evaluate whether the overall findings are robust when the analysis is limited to studies of moderate quality. In addition, another sensitivity analysis was conducted by including only studies that used objective measures for COPD diagnosis (e.g., pulmonary function tests or EHRs) to assess the impact of outcome ascertainment on the results.

### 3.4 Association between e-cigarette users and the prevalence of COPD

**Figure 2:**
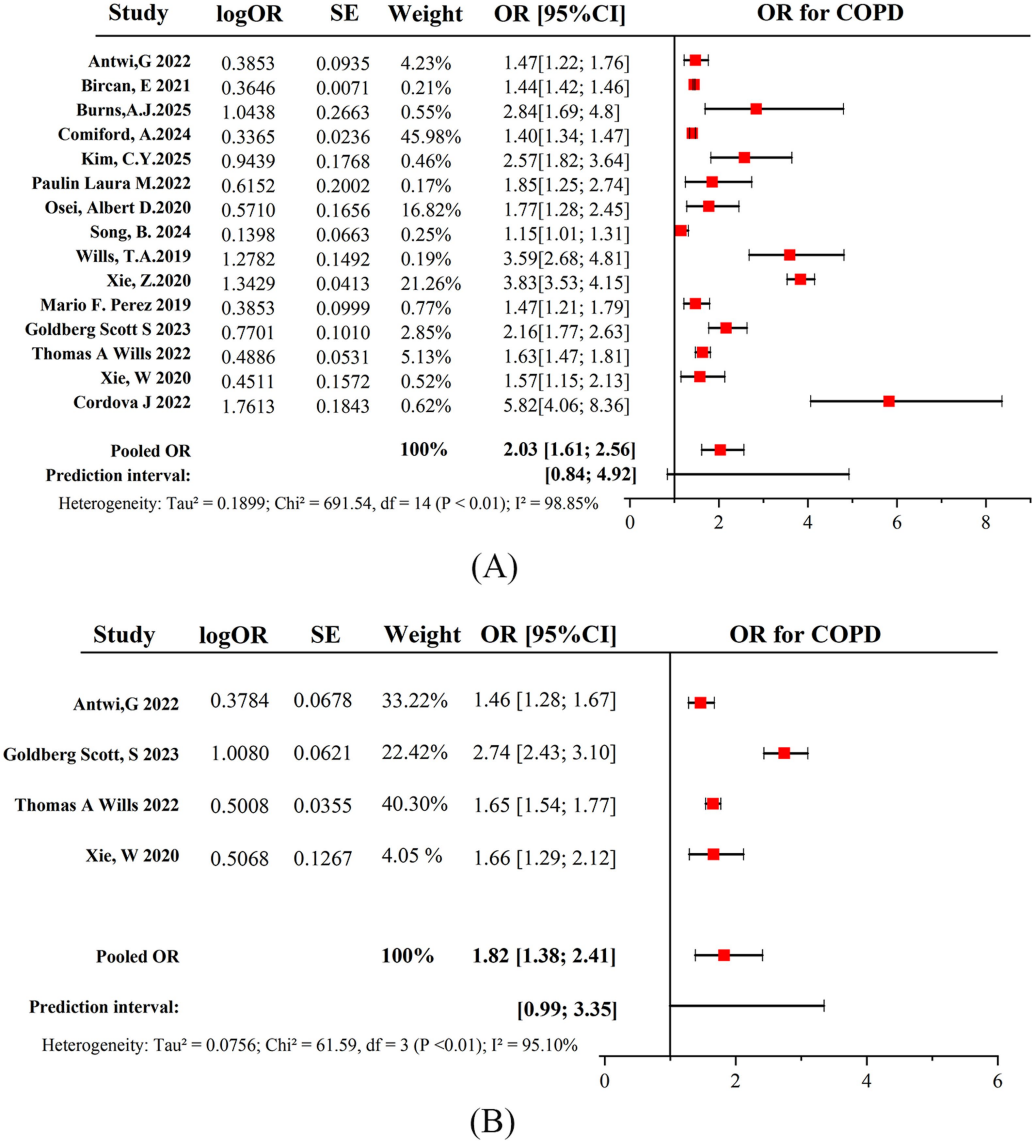
Pooled OR for greater odds of developing COPD among **(A)** current e-cigarette users and **(B)** former e-cigarette users.

The meta-analysis suggested a statistically significant association between current e-cigarette use and the increased prevalence of COPD, with a pooled OR of 2.03 (95% CI: 1.61–2.56), indicating a 103% higher prevalence compared to never-smokers, which is defined as lifelong smoking of less than 100 cigarettes or never participating in smoking or using any tobacco products, including e-cigarettes. Although all studies consistently indicated a statistically significant link between e-cigarette use and the odds of having COPD, the selected studies exhibited high heterogeneity (*I*^2^ = 98.85%), and the prediction interval for the ORs was 0.84 to 4.92 (Figure 2A).

The meta-analysis indicated a statistically significant association between former e-cigarette use and the odds of having COPD, with a pooled OR of 1.82 (95% CI: 1.38–2.41). Compared to never-smokers, former e-cigarette users had 82% higher odds of developing COPD. Although all studies consistently indicated a statistically significant link between former e-cigarette use and increased COPD prevalence, the selected studies exhibited high heterogeneity (*I*^2^ = 95.10%), and the prediction interval for the ORs was 0.99 to 3.35 (Figure 2B).

### 3.5 Association between exclusive e-cigarette users and the prevalence of COPD

**Figure 3:**
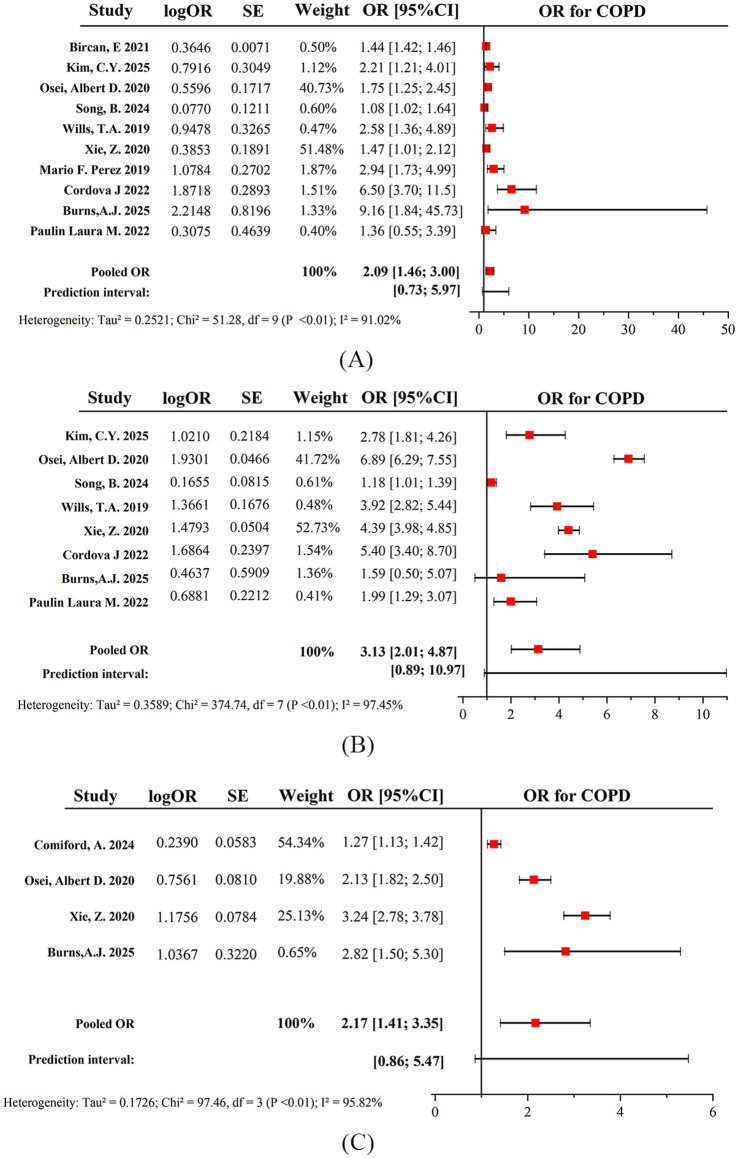
Pooled OR for greater odds of developing COPD among **(A)** exclusive e-cigarette users, **(B)** dual users, and **(C)** current e-cigarette users with a smoking history.

We then conducted a pre-specified subgroup analysis focusing on exclusive e-cigarette users, defined as individuals who currently use e-cigarettes but have never smoked traditional cigarettes. The meta-analysis suggested a statistically significant association between exclusive e-cigarette users and the odds of having COPD, with a pooled OR of 2.09 (95% CI: 1.46–3.00). Compared to never-smokers, exclusive e-cigarette users had 109% higher odds of developing COPD. Despite all studies consistently indicating a statistically significant positive association between exclusive e-cigarette use and the odds of having COPD, the selected studies exhibited high heterogeneity (*I*^2^ = 91.02%), and the prediction interval for the ORs was 0.73 to 5.97 (Figure 3A).

### 3.6 Association between dual users and the prevalence of COPD

Dual users were defined as individuals currently using both e-cigarettes and traditional cigarettes. The meta-analysis indicated a statistically significant association between dual users and the enhanced prevalence of COPD, with a pooled OR of 3.13 (95% CI: 2.01–4.87). Compared to never-smokers, dual users had 213% higher odds of developing COPD. Although most studies indicated a statistically significant positive association between dual users and the prevalence of COPD, the selected studies exhibited high heterogeneity (*I^2^* = 97.45%), and the prediction interval for the ORs ranged from 0.89 to 10.97 (Figure 3B).

### 3.7 Association between current e-cigarette users with a smoking history and the prevalence of COPD

Current e-cigarette users with a smoking history were defined as individuals who currently use e-cigarettes and have previously used traditional cigarettes but are no longer using them. The meta-analysis suggested a statistically significant association between current e-cigarette users with a smoking history and the odds of developing COPD, with a pooled OR of 2.17 (95% CI: 1.41–3.35). Compared to never-smokers, current e-cigarette users with a smoking history had 117% greater odds of having COPD. Although all studies consistently indicated a statistically significant positive association between current e-cigarette users with a smoking history and the prevalence of COPD, the included studies showed high heterogeneity (*I^2^* = 95.82%), and the prediction interval for the ORs was 0.86 to 5.47 (Figure 3C).

### 3.8 Subgroup analysis of the association between current e-cigarette users and the prevalence of COPD

**Figure 4:**
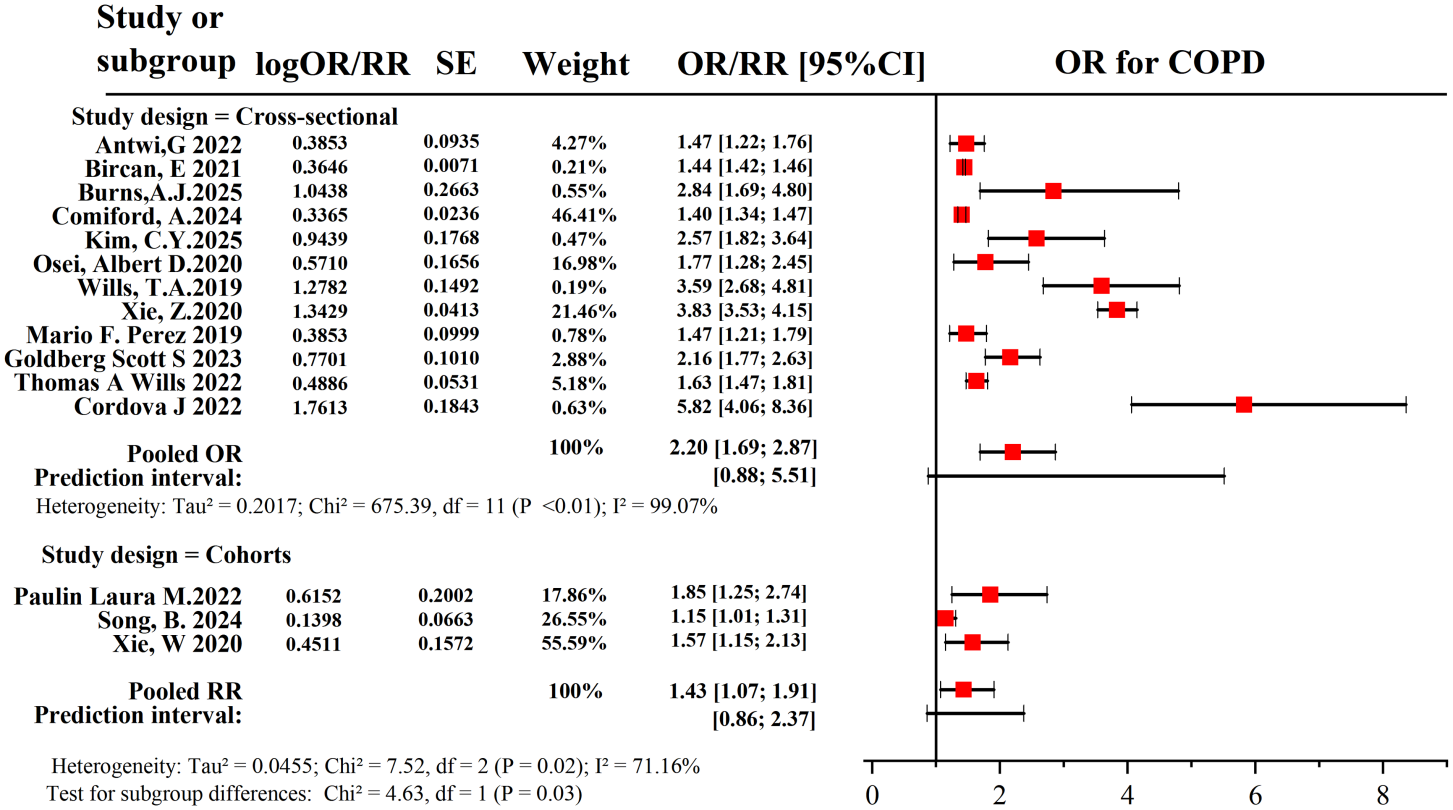
Subgroup analysis of pooled OR/RR shows the association between current e-cigarette users and greater odds of developing COPD.

Subgroup analysis was conducted based on study design (cross-sectional and prospective cohort) to explore potential sources of heterogeneity in the association between current e-cigarette use and the odds of COPD, as well as the potential impact of different effect measures. The pooled OR for the cross-sectional studies was 2.20 (95% CI: 1.69–2.87), indicating a statistically significant association. The pooled RRs for the prospective cohort studies were 1.43 (95% CI: 1.07–1.91), also indicating a statistically significant association. The heterogeneity of the ORs and RRs was 99.07 and 71.16%, respectively. The prediction intervals for ORs and RRs were 0.88 to 5.51 and 0.86 to 2.37, respectively. However, the difference in the pooled effect size indicators between the prospective cohort study and the cross-sectional study was statistically significant (*p* = 0.03 < 0.05) (Figure 4).

### 3.9 Sensitivity analysis and publication bias

This study used the leave-one-out method for sensitivity analysis to assess the robustness of the pooled estimates. The pooled ORs for current e-cigarette users ranged from 1.89 to 2.12, for former e-cigarette users from 1.59 to 1.97, for exclusive e-cigarette users from 1.73 to 2.29, for dual users from 2.75 to 3.78, and for current e-cigarette users with a smoking history from 1.85 to 2.66 (Supplementary Figure S1). Exclusion of any single study did not significantly alter the overall effect size, confirming the statistical significance of the association between e-cigarette use (across all groups) and the odds of developing COPD.

To assess the sensitivity of the pooled results to high-weight studies, the pooled ORs and heterogeneity were recalculated after excluding the three highest-weight studies (21, 27, 24). The results suggested that the pooled ORs was 2.01 (95% CI: 1.55–2.60), the heterogeneity *I^2^* was 97.29%, and the prediction interval was 0.82 to 4.88 (Supplementary Figure S2A). This exclusion did not have a substantial impact on the results, emphasizing the stability of the positive correlation between e-cigarette use and COPD prevalence.

Additionally, sensitivity analysis was performed for studies that included COPD diagnoses based on pulmonary function tests/EHRs; the pooled ORs for current e-cigarette users were 1.82 (95% CI: 1.12–2.96), the heterogeneity *I^2^* was 93.93%, and the prediction interval was 0.71 to 4.67. Sensitivity analysis was further conducted for studies with NOS score ≥5, and the pooled ORs for current e-cigarette users were 1.76 (95% CI: 1.32–2.35), the heterogeneity *I^2^* was 85.63%, and the prediction interval was 0.92 to 3.37 (Supplementary Figures S2B,C).

Publication bias was assessed using Egger’s test and funnel plot (Supplementary Figure S3). The assessment of funnel plot symmetry is inherently subjective. The quantitative Egger’s test for publication bias had a *p*-value of 0.089. It is essential to note that, given the limited number of included studies, the statistical power of Egger’s test is low. Therefore, while this result did not provide statistically significant evidence of publication bias, the potential for undetected publication bias cannot be ruled out.

## 4 Discussion

This meta-analysis, including 15 studies with approximately 4.2 million participants, suggests a significant association between e-cigarette use and increased odds of developing COPD. The pooled ORs for current e-cigarette users and former e-cigarette users were 2.03 (95% CI: 1.61–2.56) and 1.82 (95% CI: 1.38–2.41), respectively, compared to never-smokers. However, the original analysis did not fully account for the confounding effect of traditional cigarette use, which is a well-established major risk factor for COPD. Previous studies have shown that, compared with never-smokers, current smokers have an OR of 3.51 (95% CI: 3.08–3.99), and former smokers have an OR of 2.89 (95% CI: 2.63–3.17) for COPD risk, with smoking duration being a key factor (33, 34). Our subgroup analyses, which classified participants by smoking history, suggested that dual users had the highest association (OR = 3.13, 95% CI: 2.01–4.87), followed by current e-cigarette users with a smoking history (OR = 2.17, 95% CI: 1.41–3.35) and exclusive e-cigarette users (OR = 2.09, 95% CI: 1.46–3.00). Although the risk for exclusive e-cigarette users appears lower than for traditional smokers, the association remains statistically significant. Our results align with findings from Shabil et al. (35), who reported a 47% increased COPD prevalence among current e-cigarette users. However, caution is warranted, as most studies did not report on the duration or patterns of e-cigarette use, limiting the ability to draw definitive conclusions about the risks associated with long-term use.

Laboratory studies have shown that e-cigarettes can influence multiple biological processes linked to respiratory system damage and disease susceptibility, including airway inflammation, oxidative stress, and reduced lung function, all of which are central mechanisms in the pathogenesis of COPD. Therefore, despite being less harmful than traditional cigarettes, e-cigarettes are not entirely association-free. Short-term use of e-cigarettes has been shown to acutely affect airway physiology, leading to reduced blood oxygen saturation, increased airway resistance, and decreased specific airway conductance (36). However, the long-term health effects, particularly the cumulative impact on the respiratory system, remain unclear (37).

Moreover, while e-cigarettes were initially promoted as a smoking cessation tool, studies have revealed that dual use of e-cigarettes and traditional cigarettes is common among smokers attempting to quit, which may reduce the success rate of smoking cessation (38, 39). Given the already high prevalence of COPD in adults, even a moderate additional risk from e-cigarettes could have a significant public health impact. As recommended by the World Health Organization, more attention should be given to regulating the dual use of e-cigarettes and conventional cigarettes (40).

The strength of evidence varies by study design. In the subgroup analyses that included only prospective cohort studies, the pooled RRs for current e-cigarette users were 1.43 (95% CI: 1.07–1.91). Prospective cohort studies can establish the temporal sequence between e-cigarette use and COPD outcomes, providing stronger evidence for causal inference than cross-sectional studies. However, due to the limited number of prospective cohort studies, the prediction interval was 0.86 to 2.37, and the heterogeneity was high (*I^2^* = 71.16%), meta-analysis results on the association between exclusive e-cigarette use and COPD outcomes could not be obtained. Although cross-sectional studies suggested stronger cross-sectional associations (OR = 2.20, 95% CI: 1.69–2.87), the results may be influenced by inherent limitations of cross-sectional designs, particularly the inability to exclude reverse causality and survivor bias. The pooled estimated ORs from cross-sectional studies were higher than the estimated RRs from cohort studies, which is consistent with expectations. Despite numerical differences, both subgroups demonstrated a statistically significant positive correlation, supporting the core conclusion of an association between e-cigarette use and the prevalence of COPD. This underscores the critical need for more prospective cohort studies focused on e-cigarette use and COPD risk.

In a sensitivity analysis using studies with pulmonary function tests or EHR data to diagnose COPD, the pooled ORs were 1.82 (95% CI: 1.12–2.96), though this result was based on only three studies with high heterogeneity. This highlights the necessity for high-quality research using objective diagnostic criteria to more reliably assess the link between e-cigarette use and COPD. The observed risk increase in e-cigarette users aligns with previous findings that e-cigarettes negatively affect pulmonary function and airway inflammation, even among never-smokers (41).

The extremely high statistical heterogeneity observed in this meta-analysis (*I^2^* = 98.85%) indicates differences among the included studies. In this situation, the pooled ORs are highly dependent on the studies with the greatest weight. In our analysis, three studies (21, 27, 24) contributed approximately 84% of the total weight, suggesting that the pooled results may largely reflect the characteristics of these studies rather than a consistent effect across all research. Nevertheless, a sensitivity analysis excluding these three studies yielded an OR of 2.01 (95% CI: 1.55–2.60), reinforcing a positive association. Meanwhile, high statistical heterogeneity fundamentally impacted the interpretation of our study results. The broad prediction interval that includes the null value (OR = 1) indicates that the true effect in new settings could vary significantly. Therefore, the pooled ORs should be interpreted as an average of varying estimates, not a precise or universally applicable measure.

Furthermore, although the studies included in this meta-analysis have statistically adjusted for key covariates, including age, gender, race, education level, and so on, and conducted subgroup analysis based on traditional smoking history, thereby strengthening the inference of an association between exclusive e-cigarette use and COPD prevalence, the possibility of residual confounding still exists. Adjustment of statistical models is not equivalent to restriction methods based on study design and may not fully capture the complex and dose-dependent effects of past smoking history. In addition, differences in the definition, measurement, and adjustment methods of smoking history across studies not only introduce heterogeneity but may also lead to incomplete control of certain confounding factors. Therefore, the observed association, despite multivariable adjustment, may still be partially influenced by unmeasured or imperfectly measured residual factors related to smoking or other health behaviors. Future research should further clarify and quantify these potential residual confounding factors through more precise measurements, unified standards, stricter restrictions, or longer follow-up periods in the study design. This consideration is also consistent with the increasing evidence that the harm of e-cigarettes to respiratory health, especially its long-term effects, may be more significant than previously recognized (42).

Despite the valuable insights this study provides into the link between e-cigarette use and COPD prevalence, there are several limitations. First, the included studies exhibited significant variability in design, e-cigarette product types, usage patterns, and population characteristics. Although a random-effects model was used to account for heterogeneity, the high level of heterogeneity suggests that further studies are needed to clarify these issues. Therefore, the pooled ORs obtained in this study should be interpreted with caution, as it represents a weighted average under the current, uneven distribution of evidence. Second, most studies relied on self-reported COPD diagnoses, which may introduce recall bias or misdiagnosis bias, affecting the accuracy of the results. Critically, only 3 of the 15 included studies utilized objective diagnostic criteria. Although the sensitivity analysis result indicates that the subgroups using objective diagnostic criteria remain statistically significant, their extremely small number remains a major limitation. Third, key exposure data—such as duration of use, frequency of use, and e-liquid composition—were unavailable, limiting the ability to analyze dose–response relationships and cumulative risk. Fourth, while most studies adjusted for covariates such as age, the lack of individual-level data hindered a complete understanding of the complex interactions between age, e-cigarette use, and COPD prevalence. Fifth, 86% of the studies were from the United States, with only one each from South Korea and China’s Hebei Province, limiting the generalizability of findings. There is a critical need for studies in other regions with diverse ethnicities, e-cigarette policies, and healthcare systems. Finally, 80% of studies were observational, so this meta-analysis could only identify associations, not establish causality. Large-scale, prospective cohort studies are required to explore the temporal sequence and potential causal pathways between e-cigarette use and COPD.

## 5 Conclusion

This meta-analysis indicates that current e-cigarette use is associated with an increased prevalence of COPD. Importantly, this association remained significant in the subgroup analysis of exclusive e-cigarette users, suggesting a potential link independent of traditional smoking. Clinicians should consider monitoring and evaluating the respiratory health of e-cigarette users, particularly those who also use traditional cigarettes. It is suggested that public health measures focus on increasing awareness of the potential respiratory risks of electronic cigarettes, promoting safer behavioral norms, and ultimately improving the overall respiratory health status of the population. Future research should prioritize rigorous longitudinal studies, with more detailed exposure data, to clarify the long-term effects of e-cigarette use and establish causal relationships with COPD.

## Data Availability

The data analyzed in this study is subject to the following licenses/restrictions: The datasets used and/or analysed during the current study are available from the corresponding author on reasonable request. Requests to access these datasets should be directed to yangmengya40@163.com.
