# Supplementary material for: Association between current e-cigarette use and chronic obstructive pulmonary disease: a meta-analysis focusing on exclusive e-cigarette users
Source: Front Public Health. 2026 Apr 22;14:1802572. doi: 10.3389/fpubh.2026.1802572 (PMC13143967; doi:10.3389/fpubh.2026.1802572)
Supplement: Supplementary file 1 [file Data_Sheet_1.docx]

***Supplementary Material***

**Table S1.** PRISMA Checklist

| **Section and Topic** | **Item #** | **Checklist item** | **Location where item is reported** |
| --- | --- | --- | --- |
| **TITLE** | | |  |
| Title | 1 | Identify the report as a systematic review. | Title |
| **ABSTRACT** | | |  |
| Abstract | 2 | See the PRISMA 2020 for Abstracts checklist. (made as per the Journal guidelines) | Abstract |
| **INTRODUCTION** | | |  |
| Rationale | 3 | Describe the rationale for the review in the context of existing knowledge. | 1.Introduction |
| Objectives | 4 | Provide an explicit statement of the objective(s) or question(s) the review addresses. | 1.Introduction |
| **METHODS** | | |  |
| Eligibility criteria | 5 | Specify the inclusion and exclusion criteria for the review and how studies were grouped for the syntheses. | 2.3 Eligibility criteria |
| Information sources | 6 | Specify all databases, registers, websites, organisations, reference lists and other sources searched or consulted to identify studies. Specify the date when each source was last searched or consulted. | 2.2 Data sources and search strategy |
| Search strategy | 7 | Present the full search strategies for all databases, registers and websites, including any filters and limits used. | 2.2 Data sources and search strategy, Table S2 |
| Selection process | 8 | Specify the methods used to decide whether a study met the inclusion criteria of the review, including how many reviewers screened each record and each report retrieved, whether they worked independently, and if applicable, details of automation tools used in the process. | 2.4 Study selection |
| Data collection process | 9 | Specify the methods used to collect data from reports, including how many reviewers collected data from each report, whether they worked independently, any processes for obtaining or confirming data from study investigators, and if applicable, details of automation tools used in the process. | 2.5 Data extraction and quality assessment |
| Data items | 10a | List and define all outcomes for which data were sought. Specify whether all results that were compatible with each outcome domain in each study were sought (e.g., for all measures, time points, analyses), and if not, the methods used to decide which results to collect. | 2.5 Data extraction and quality assessment, Table 1 |
|  | 10b | List and define all other variables for which data were sought (e.g., participant and intervention characteristics, funding sources). Describe any assumptions made about any missing or unclear information. | 2.5 Data extraction and quality assessment, Table 1 |
| Study risk of bias assessment | 11 | Specify the methods used to assess risk of bias in the included studies, including details of the tool(s) used, how many reviewers assessed each study and whether they worked independently, and if applicable, details of automation tools used in the process. | 2.5 Data extraction and quality assessment, Table S3 |
| Effect measures | 12 | Specify for each outcome the effect measure(s) (e.g. risk ratio, mean difference) used in the synthesis or presentation of results. | 2.6 Data synthesis and statistical analysis |
| Synthesis methods | 13a | Describe the processes used to decide which studies were eligible for each synthesis (e.g. tabulating the study intervention characteristics and comparing against the planned groups for each synthesis (item #5)). | 3.3 Quality assessment of included studies, Table 1 |
|  | 13b | Describe any methods required to prepare the data for presentation or synthesis, such as handling of missing summary statistics, or data conversions. | NA |
|  | 13c | Describe any methods used to tabulate or visually display results of individual studies and syntheses. | 2.6 Data synthesis and statistical analysis |
|  | 13d | Describe any methods used to synthesize results and provide a rationale for the choice(s). If meta-analysis was performed, describe the model(s), method(s) to identify the presence and extent of statistical heterogeneity, and software package(s) used. | 2.6 Data synthesis and statistical analysis |
|  | 13e | Describe any methods used to explore possible causes of heterogeneity among study results (e.g. subgroup analysis, meta-regression). | 2.6 Data synthesis and statistical analysis |
|  | 13f | Describe any sensitivity analyses conducted to assess robustness of the synthesized results. | 2.6 Data synthesis and statistical analysis |
| Reporting bias assessment | 14 | Describe any methods used to assess risk of bias due to missing results in a synthesis (arising from reporting biases). | NA |
| Certainty assessment | 15 | Describe any methods used to assess certainty (or confidence) in the body of evidence for an outcome. | NA |
| **RESULTS** | | |  |
| Study selection | 16a | Describe the results of the search and selection process, from the number of records identified in the search to the number of studies included in the review, ideally using a flow diagram. | 3.1 Literature search, Figure 1 |
|  | 16b | Cite studies that might appear to meet the inclusion criteria, but which were excluded, and explain why they were excluded. | 3.1 Literature search, Figure 1 |
| Study characteristics | 17 | Cite each included study and present its characteristics. | Table 1 |
| Risk of bias in studies | 18 | Present assessments of risk of bias for each included study. | Table S3 |
| Results of individual studies | 19 | For all outcomes, present, for each study: (a) summary statistics for each group (where appropriate) and (b) an effect estimate and its precision (e.g. confidence/credible interval), ideally using structured tables or plots. | Table 1 |
| Results of syntheses | 20a | For each synthesis, briefly summarise the characteristics and risk of bias among contributing studies. | 3.2 Characteristics of included studies - 3.3 Quality assessment of included studies |
|  | 20b | Present results of all statistical syntheses conducted. If meta-analysis was done, present for each the summary estimate and its precision (e.g. confidence/credible interval) and measures of statistical heterogeneity. If comparing groups, describe the direction of the effect. | 3.4 Association between e-cigarette users and the prevalence of COPD - 3.9 Sensitivity analysis and publication bias |
|  | 20c | Present results of all investigations of possible causes of heterogeneity among study results. | 3.8 Subgroup analysis of association between current e-cigarettes users and the prevalence of COPD - 3.9 Sensitivity analysis and publication bias |
|  | 20d | Present results of all sensitivity analyses conducted to assess the robustness of the synthesized results. | 3.9 Sensitivity analysis and publication bias |
| Reporting biases | 21 | Present assessments of risk of bias due to missing results (arising from reporting biases) for each synthesis assessed. | NA |
| Certainty of evidence | 22 | Present assessments of certainty (or confidence) in the body of evidence for each outcome assessed. | NA |
| **DISCUSSION** | | |  |
| Discussion | 23a | Provide a general interpretation of the results in the context of other evidence. | 4. Discussion |
|  | 23b | Discuss any limitations of the evidence included in the review. | 4. Discussion |
|  | 23c | Discuss any limitations of the review processes used. | 4. Discussion |
|  | 23d | Discuss implications of the results for practice, policy, and future research. | 5. Conclusions |
| **OTHER INFORMATION** | | |  |
| Registration and protocol | 24a | Provide registration information for the review, including register name and registration number, or state that the review was not registered. | 2.1 Study design |
|  | 24b | Indicate where the review protocol can be accessed, or state that a protocol was not prepared. | 2.1 Study design |
|  | 24c | Describe and explain any amendments to information provided at registration or in the protocol. | NA |
| Support | 25 | Describe sources of financial or non-financial support for the review, and the role of the funders or sponsors in the review. | Funding |
| Competing interests | 26 | Declare any competing interests of review authors. | Conflict of Interest |
| Availability of data, code and other materials | 27 | Report which of the following are publicly available and where they can be found: template data collection forms; data extracted from included studies; data used for all analyses; analytic code; any other materials used in the review. | Data Availability Statement |

**Table S2.** The adjusted search terms as per searched electronic databases

| Database | Search Query | Results |
| --- | --- | --- |
|  | | |
|  | | |
| PubMed | #1 ("Electronic Nicotine Delivery Systems"[Mesh] OR "Vaping"[Mesh] )  #2(e-cigarette*[tiab] OR "ecigarette*"[tiab] OR "electronic cigarette*"[tiab] OR "electronic nicotine delivery system*"[tiab] OR "ENDS"[tiab] OR vape[tiab] OR vapes[tiab] OR vaping[tiab] OR "e-liquid*"[tiab] OR "e juice"[tiab])  #3 #1 OR #2  #4 "Pulmonary Disease, Chronic Obstructive"[Mesh]  #5 (COPD[tiab] OR "chronic obstructive pulmonary disease"[tiab] OR "chronic obstructive lung disease"[tiab] OR "chronic obstructive airway disease"[tiab] OR emphysema[tiab] OR "chronic bronchitis"[tiab])  #6 #4 OR #5  #7 #3 AND #6 | **368** |
|  | | |
| Embase | ('electronic cigarette'/exp OR 'vaping'/exp) OR (e-cigarette*:ti,ab OR ecigarette*:ti,ab OR 'electronic cigarette*':ti,ab OR 'electronic nicotine delivery system*':ti,ab OR ends:ti,ab OR vape:ti,ab OR vapes:ti,ab OR vaping:ti,ab OR 'e-liquid*':ti,ab OR 'e juice':ti,ab)  AND  ('chronic obstructive lung disease'/exp OR 'emphysema'/exp OR 'chronic bronchitis'/exp) OR (copd:ti,ab OR 'chronic obstructive pulmonary disease':ti,ab OR 'chronic obstructive lung disease':ti,ab OR 'chronic obstructive airway disease':ti,ab OR emphysema:ti,ab OR 'chronic bronchitis':ti,ab) | 1103 |
|  | | |
| Web of Science | (TS=("Electronic Nicotine Delivery Systems" OR "Vaping" OR "E-Cigarettes")) OR (TS=(e-cigarette* OR ecigarette* OR "electronic cigarette*" OR "electronic nicotine delivery system*" OR ENDS OR vape OR vapes OR vaping OR "e-liquid*" OR "e juice*"))  AND  (TS=("Pulmonary Disease, Chronic Obstructive" OR "COPD" OR "chronic obstructive pulmonary disease" OR "chronic obstructive lung disease" OR "chronic obstructive airway disease" OR emphysema OR "chronic bronchitis")) | 5931 |
| Cochrane Library | (MeSH descriptor: [Electronic Nicotine Delivery Systems] explode all trees) OR (MeSH descriptor: [Vaping] explode all trees) OR (MeSH descriptor: [E-Cigarettes] explode all trees) OR (e-cigarette* OR ecigarette* OR "electronic cigarette*" OR "electronic nicotine delivery system*" OR ENDS OR vape OR vapes OR vaping OR "e-liquid*" OR "e juice"):ti,ab,kw  AND  (MeSH descriptor: [Pulmonary Disease, Chronic Obstructive] explode all trees) OR (COPD OR "chronic obstructive pulmonary disease" OR "chronic obstructive lung disease" OR "chronic obstructive airway disease" OR emphysema OR "chronic bronchitis"):ti,ab,kw | 62 |
| China National Knowledge Infrastructure | (SU=('电子烟' OR '电子尼古丁传递系统' OR 'ENDS' OR '蒸汽烟' OR ' vaping' OR 'e-cigarette' OR 'ecigarette' OR 'e-liquid' OR '烟油') OR TI=('电子烟' OR '电子尼古丁传递系统' OR 'ENDS' OR '蒸汽烟') OR KY=('电子烟' OR '蒸汽烟')) AND (SU=('慢性阻塞性肺疾病' OR '慢阻肺' OR 'COPD' OR '肺气肿' OR '慢性支气管炎') OR TI=('慢性阻塞性肺疾病' OR '慢阻肺' OR 'COPD') OR KY=('慢性阻塞性肺疾病' OR '慢阻肺' OR 'COPD')) | 3 |
| Wanfang Patent Database | ( (主题:("电子烟" OR "电子尼古丁传递系统" OR "ENDS" OR "蒸汽烟" OR "vaping" OR "e-cigarette" OR "ecigarette" OR "electronic cigarette" OR "electronic nicotine delivery system" OR "e-liquid" OR "烟油")) OR (题名:("电子烟" OR "蒸汽烟")) OR (关键词:("电子烟" OR "蒸汽烟")) ) AND ( (主题:("慢性阻塞性肺疾病" OR "慢阻肺" OR "COPD" OR "肺气肿" OR "慢性支气管炎")) OR (题名:("慢性阻塞性肺疾病" OR "慢阻肺" OR "COPD")) OR (关键词:("慢性阻塞性肺疾病" OR "慢阻肺" OR "COPD")) ) | 136 |

**Table S3.** Quality assessment using Newcastle-Ottawa Scale

| **STUDY** | **SELECTION (max 4 points)** | | | | **COMPARABILITY (max 2 points)** | **OUTCOME (max 3 points)** | | | **SCORE (out of 9 for cohort/6 for cross-sectional)** |
| --- | --- | --- | --- | --- | --- | --- | --- | --- | --- |
|  | Representativeness | Selection | Ascertainment | Demonstration of the outcome of interest was not present at start of study | Comparability the basis of the design or analysis | Assessment of outcome | Was follow-up long enough for outcomes to occur? | Adequacy of the follow-up |  |
| Antwi GO 2022 | 1 | 1 | NA | NA | 2 | NA | NA | NA | 4 |
| Bircan E 2021 | 1 | 1 | NA | NA | 2 | NA | NA | NA | 4 |
| Burns AJ 2025 | 1 | 1 | NA | NA | 2 | NA | NA | NA | 4 |
| Comiford A  2024 | 1 | 1 | NA | NA | 2 | NA | NA | NA | 4 |
| Kim CY 2025 | 1 | 1 | NA | NA | 2 | 1 | NA | NA | 5 |
| Paulin LM 2022 | 1 | 1 | 1 | NA | 2 | NA | NA | NA | 5 |
| Osei AD 2020 | 1 | 1 | NA | NA | 2 | NA | NA | NA | 4 |
| Song B 2024 | NA | 1 | NA | 1 | 2 | 1 | NA | NA | 5 |
| Wills TA 2019 | NA | 1 | NA | NA | 2 | NA | NA | NA | 3 |
| Xie Z 2020 | 1 | 1 | NA | NA | 2 | NA | NA | NA | 4 |
| Perez MF 2019 | 1 | 1 | NA | NA | 2 | NA | NA | NA | 4 |
| Goldberg Scott S 2023 | 1 | 1 | NA | NA | 2 | 1 | NA | NA | 5 |
| Wills TA 2022 | 1 | 1 | NA | NA | 2 | NA | NA | NA | 4 |
| Xie W 2020 | 1 | 1 | NA | 1 | 2 | 1 | NA | NA | 6 |
| Cordova J 2022 | 1 | 1 | NA | NA | 2 | NA | NA | NA | 4 |


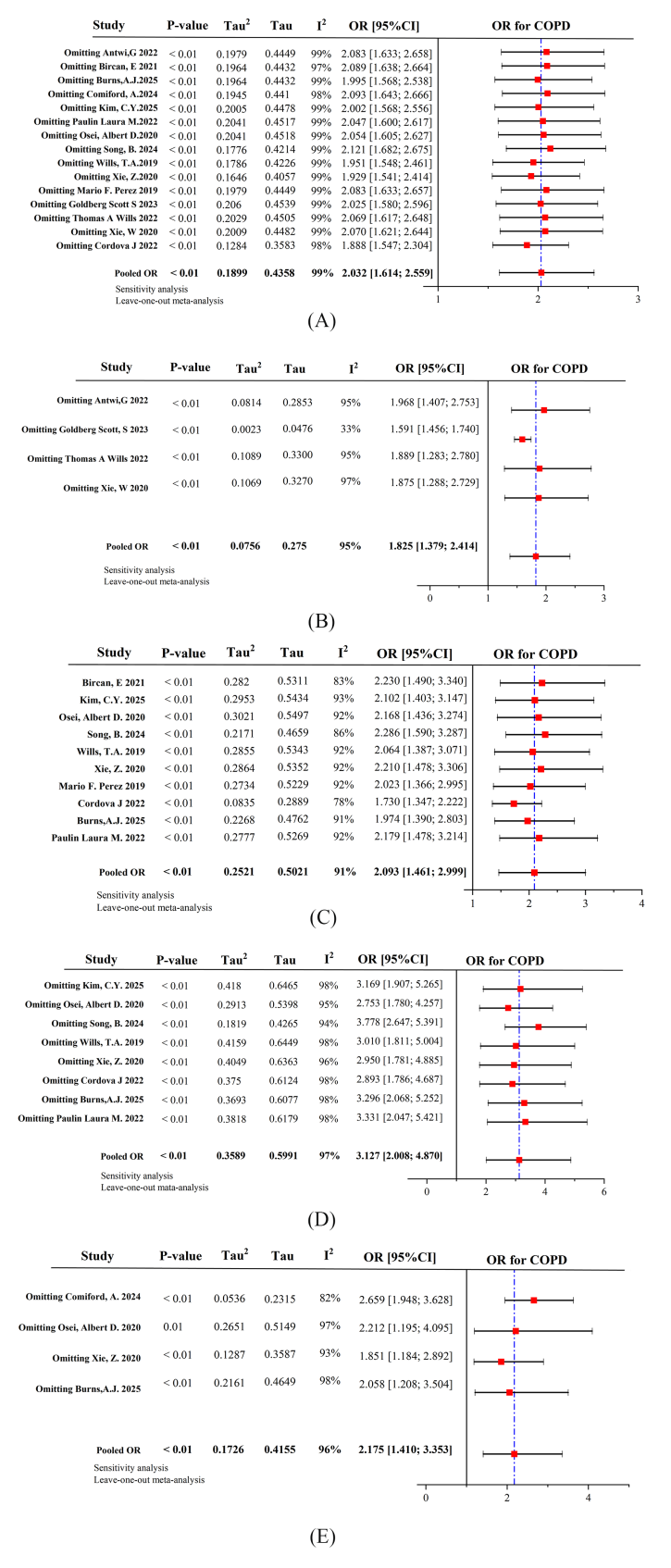


Figure S1 Sensitivity analysis of pooled OR showing the association between (A) current e-cigarette users, (B) former e-cigarette users, (C) exclusive e-cigarette users, (D) dual user, (E) current e-cigarette users with a smoking history and greater odds of having COPD


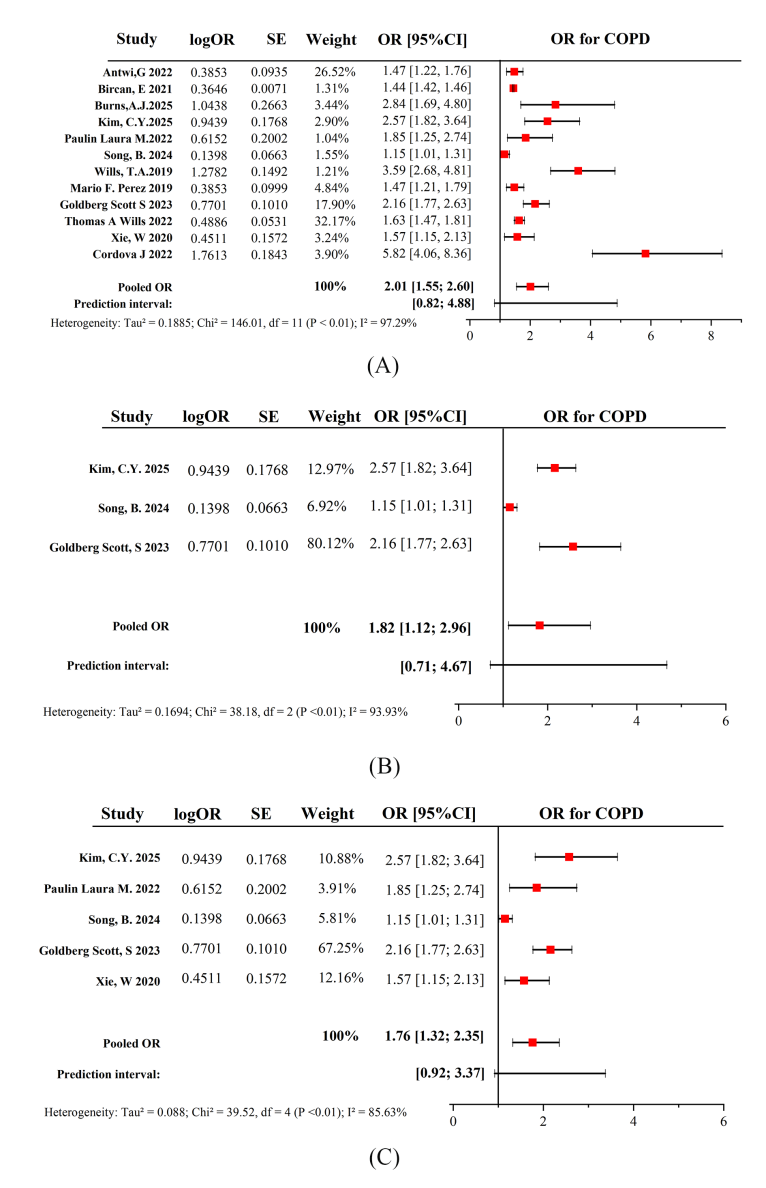


Figure S2 Sensitivity analysis (A) after excluding the top 3 studies with the highest weights, (B) only including studies with COPD diagnosis based on spirometry or electronic health record data, (C) only including studies with a NOS score≥5.


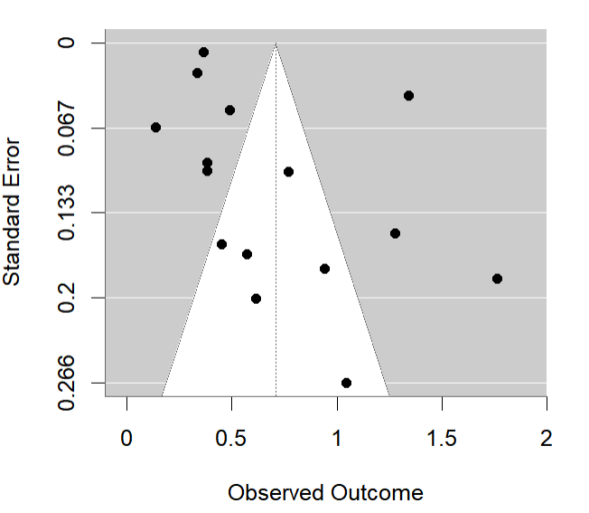


Figure S3 Funnel plot analysis of pooled OR showing the association between current e-cigarettes users and greater odds of having COPD
